# Lack of association between COMT gene and deficit/nondeficit schizophrenia

**DOI:** 10.1186/1744-9081-2-42

**Published:** 2006-12-15

**Authors:** Ikwunga Wonodi, Braxton D Mitchell, O Colin Stine, L Elliot Hong, Amie Elliott, Brian Kirkpatrick, William T Carpenter, Gunvant K Thaker, Robert W Buchanan

**Affiliations:** 1 Maryland Psychiatric Research Center, Department of Psychiatry, University of Maryland School of Medicine, P.O. Box 21247, Baltimore, Maryland, 21228, USA; 2 General Clinical Research Center (GCRC) Genomics Core Facility, University of Maryland School of Medicine, Baltimore, Maryland, USA; 3 Department of Medicine, University of Maryland School of Medicine, Baltimore, Maryland, USA

## Abstract

**Background:**

The dopamine dysregulation hypothesis of schizophrenia posits that positive, negative and cognitive symptoms correlate with cortical/subcortical imbalances in dopaminergic transmission. A functional polymorphism (Val^158^Met) in the catechol-*O*-methyltransferase (COMT) gene is implicated in the pathophysiology of schizophrenia by its effect on prefrontal dopamine transmission, and its unique impact on prefrontal cognitive and behavioral phenotypes. Cognitive impairments and negative symptoms in schizophrenia have been hypothesized to be associated with hypodopaminergic states. Schizophrenia patients with the deficit syndrome are characterized by primary enduring negative symptoms, impairment on neurocognitive tasks sensitive to frontal and parietal cortical functioning, and poorer functional outcome compared to non-deficit patients.

**Methods:**

Eighty-six schizophrenia cases that met DSM-IV criteria for schizophrenia were recruited. Additional categorization into deficit and nondeficit syndrome was performed using the Schedule for the Deficit Syndrome (SDS). A healthy comparison group (n = 50) matched to cases on age and ethnicity was recruited. Allele and genotype frequencies of the Val^158^Met polymorphism were compared among healthy controls, and schizophrenia cases with the deficit (n = 21), and nondeficit syndrome (n = 65).

**Results:**

There was a significant difference in Val/Val genotype frequencies between schizophrenia cases (combined deficit/nondeficit) and healthy controls (*p *= 0.004). No significant differences in allele or genotype frequencies were observed between deficit and nondeficit cases.

**Conclusion:**

Results from this preliminary analysis failed to show an effect of COMT gene on deficit schizophrenia.

## Background

Schizophrenia is a complex phenotype with a multifactorial etiology. Reducing heterogeneity by identifying alternative phenotypes or valid subtypes that reflect specific neurobiological processes in the pathophysiology of the disorder is critical to uncovering susceptibility genes [1-3]. The concept of the deficit syndrome subtype of schizophrenia arose in an attempt to reduce clinical heterogeneity [4-6]. The deficit syndrome is characterized by primary (idiopathic) and enduring negative symptoms that persist through epochs of clinical stability and decompensation. By definition, idiopathic negative symptoms are not attributable to paranoid or psychotic withdrawal, depression, anxiety, or medication side effects. Schizophrenia patients who do not meet the deficit syndrome criteria are classified as nondeficit. Compelling evidence supports the construct validity of the deficit/nondeficit subgrouping on clinical features and prognosis, risk factors, neurocognitive and biological correlates, and pharmacological response profiles, and that dysfunction in the neural circuit that includes the dorsolateral prefrontal cortex (DLPFC) form the neuropathological basis for the deficit syndrome [6,7].

The reformulated dopamine hypothesis of schizophrenia describes interdependent prefrontal hypodopaminergia (associated with negative and cognitive symptoms) and subcortical hyperdopaminergia (associated with positive symptoms) [8]. The COMT gene is implicated in the pathophysiology of schizophrenia by its effect on prefrontal dopamine. A functional single nucleotide polymorphism (SNP) (Val^158^Met) of COMT gene results in two common variants, Valine (Val) and methionine (Met), which correspond to low and high synaptic dopamine respectively [9]. Associations between the Val^158^Met SNP and cognitive phenotypes have been described in healthy and schizophrenia individuals [10-12].

The COMT gene maps to chromosome 22q11.2, a region implicated in schizophrenia linkage studies, and in velocardiofacial syndrome (VCFS), which is characterized by psychosis and cognitive deficits [13,14]. COMT is thought to exert a distinctive effect on prefrontal dopamine-related information processing because it is primarily expressed in prefrontal cortical neurons [15,16], and is the principal enzyme in the degradation of dopamine (up to 60%) in the DLPFC; a role supported by data from animal studies [17]. Furthermore, the dopamine transporter, another key dopamine metabolizing enzyme in the brain is less expressed in the prefrontal cortex [18]. Deficit schizophrenia is characterized by impairments in tasks sensitive to frontal and parietal cortical functioning [19]. We have previously demonstrated association between COMT ^158 ^Val allele and schizophrenia in a biracial cohort of patients from the Baltimore Metropolitan area [20]. In the current report, we have extended our previous study to evaluate the association between the variant Val allele and deficit/nondeficit schizophrenia. We hypothesized that the Val allele would occur more frequently in deficit than in nondeficit schizophrenia.

## Materials and methods

Eighty-six unrelated schizophrenia individuals were recruited from the Maryland Psychiatric Research Center (MPRC). Healthy controls (n = 50) were recruited through newspaper advertisements targeting the same geographic region. All participants gave written informed consent, which was approved by the University of Maryland Institutional Review Board. Additional evaluation of the capacity to sign consent was performed on patients to assess their understanding of the study [21]. As in our previous study, there was no COMT genotype or allele association in African-American subjects, so this group was not included in the current study (n = 80). Patients met the Structured Clinical Interview for DSM-IV (SCID-IV) [22] criteria for schizophrenia or schizoaffective disorder. Additional categorization into deficit (n = 21), and nondeficit syndrome (n = 65) was performed with the Schedule for the Deficit Syndrome (SDS). For the diagnosis of deficit schizophrenia, this instrument requires the presence of two or more idiopathic and enduring negative symptoms (Kirkpatrick et al, 1989). Inter-rater reliabilities among the clinical interviewers were above 0.80 (interclass correlation). The Structured Interview for DSM-IV Personality Diagnoses (SIDP) [23], and Family History-Research Diagnostic Criteria (FH-RDC) [24] were administered in controls. Healthy controls had no Axis I diagnoses, or schizophrenia spectrum personality disorders.

Genotyping methods are described elsewhere [20]. Briefly, venous whole blood was collected and DNA isolated by standard means. COMT Val^158^Met SNP was determined as a restriction fragment length polymorphism (RFLP) after PCR amplification according to the method of Egan et al. (2001).

Fisher's exact test was used to evaluate whether the observed distribution of COMT Val^158^Met genotypes was consistent with that expected under Hardy Weinberg equilibrium. Nominal logistic regression (with age and sex as predictor variables) was used to determine the effect of genotype on disease status under an additive genetic model (i.e., by assigning values of -1, 0, and 1 to the three genotype classes). We first evaluated the effect of genotype on the total schizophrenia endpoint, combining the deficit and nondeficit subtypes. We then evaluated genotype effects on the deficit only schizophrenia endpoint.

## Results

The sample consists of 136 European-American subjects (21 deficit cases, 65 nondeficit cases, and 50 healthy controls) presented in Table 1. The observed COMT genotype distributions were consistent with that expected under Hardy Weinberg equilibrium. Comparisons of COMT genotype frequencies between cases and controls are presented in Table 1. COMT genotype was significantly associated with schizophrenia (deficit and nondeficit combined; age- and sex-adjusted Odds Ratio = 3.24, p = 0.004 by genotype test), with Val allele frequencies of 0.68 and 0.43, in cases and controls, respectively. In contrast, genotype (and allele) frequencies did not differ between deficit and nondeficit cases (allele frequencies 0.74 and 0.66, respectively; age- and sex-adjusted p = 0.8). Deficit schizophrenia was observed in one female: COMT genetic variation has been previously shown to produce sexual dimorphism in brain dopamine levels; we repeated the analyses comparing COMT genotype between deficit and nondeficit subtypes in males only. We again observed that genotype (and allele) frequencies did not differ between both groups (allele frequencies 0.72 and 0.58, respectively; p = 0.19). Given the small sample size, we estimated the least detectable difference in allele frequencies that could have been observed in our sample between deficit and nondeficit cases. Given the observed Val allele frequency of 0.66 in nondeficit cases, the frequency in the deficit group would have had to have been less than 0.42 or greater than 0.87 in order to detect a significant difference between groups (at alpha = 0.05) with 80% power.

**Table 1:** Demographic information and COMT genotype

		Schizophrenia Subjects			Healthy controls	Total
				
Sex		Nondeficit	Deficit	Total patients		
Female	N	28	1	29	22	51
	Age Mean (SD)	47.08 (8.94)	55 (0.0)	47.36 (8.91)	44.31 (17.59)	46.04 (13.29)
	Val-Val	16	1	17	6	23
	Val-Met	11	0	11	10	21
	Met-Met	1	0	1	6	7
						
Male	N	37	20	57	28	85
	Age Mean (SD)	43.80 (10.14)	43.74 (11.12)	43.78 (10.40)	46.89 (17.87)	44.80 (13.30)
	Val-Val	14	10	24	3	27
	Val-Met	15	9	24	15	39
	Met-Met	8	1	9	10	19
						
Total	N	65	21	86	50	136
	Age Mean (SD)	45.21 (9.71)	44.28 (11.12)	44.98 (10.01)	45.76 (17.62)	45.27 (13.26)
	Val-Val	30	11	41	9	50
	Val-Met	26	9	35	25	60
	Met-Met	9	1	10	16	26

N = number

SD = standard deviation

Val = Valine variant allele

Met = Methionine variant allele

COMT = catechol-*O*-methyltransferase

## Discussion

In this report we did not observe a significant difference between deficit and nondeficit schizophrenia subtypes in either COMT allele or genotype frequencies. Our report however provides an extension of our earlier finding of association between COMT^158^Val allele and schizophrenia in a larger sample of European-American subjects with the addition of deficit schizophrenia individuals. Similar to our prior report, we failed to find association between COMT gene and schizophrenia in the African-American subgroup. This suggests that COMT allele frequencies might be different in both ethnic groups in this Baltimore sample. To avoid potential population substratification, we did not include African-Americans in the current report. In our previous study based on 61 European-Americans with schizophrenia, we observed allele frequencies of 0.62 and 0.50 in cases and healthy controls (*p *= 0.04). The differences in allele frequencies persisted, and indeed increased in this study on a modestly expanded sample (to 0.680 vs. 0.430), which includes 86 European-American schizophrenia cases presented in this report. Evidence from family-based and case-control studies generally suggest that COMT Val^158^Met polymorphism might be associated with schizophrenia [25] with some negative reports [26,27].

The main limitation of this study is the small sample size, which may have limited detecting associations of only modest size between COMT gene and deficit/nondeficit subtypes. The differences are so small that the power needed to detect a significant difference between both subtypes would indeed require a large sample. However, our findings are unlikely due to type II error. Our reported allele and genotype frequencies were similar between the deficit and nondeficit schizophrenia subgroups, but considerably higher than the frequencies observed in healthy controls. In this sample, the patient group is comprised of a significantly higher proportion of males than the control group. Furthermore, there are substantially more males in the deficit group, which is consistent with prior reports on deficit schizophrenia (Kirkpatrick et al, 2001). Prior studies have reported a sexual dimorphism in COMT-associated dopamine levels on several neuropsychiatric phenotypes in a region-specific manner, particularly in the frontal cortex [28,29]. Analyses using a regression model controlled for age and sex effects on our COMT findings in this Baltimore sample.

## Conclusion

The results do not support a differential COMT gene effect on the deficit/nondeficit subtypes of schizophrenia. Future studies in larger independent samples investigating joint interactions with other candidate dopamine system genes (e.g. dopamine transporter gene [DAT1]) and COMT gene on schizophrenia endophenotypes are warranted.

## Competing interests

The author(s) declare that they have no competing interests.

## Authors' contributions

IW, OCS, and RWB collected and analyzed the data for this study, with assistance from LEH, GKT, BK, and WTC. BDM and AE assisted with statistical analyses. All authors contributed intellectually to the design of this study. All authors read and approved the final manuscript.
